# Inhibition of Arginase 1 Liberates Potent T Cell Immunostimulatory Activity of Human Neutrophil Granulocytes

**DOI:** 10.3389/fimmu.2020.617699

**Published:** 2021-02-26

**Authors:** Verena Vonwirth, Yagmur Bülbül, Anke Werner, Hakim Echchannaoui, Johannes Windschmitt, Alice Habermeier, Sonia Ioannidis, Niu Shin, Roland Conradi, Matthias Bros, Stefan Tenzer, Matthias Theobald, Ellen Ildicho Closs, Markus Munder

**Affiliations:** ^1^ Third Department of Medicine (Hematology, Oncology and Pneumology), University Medical Center of the Johannes Gutenberg University Mainz, Mainz, Germany; ^2^ German Cancer Consortium (DKTK), Partner Site Frankfurt/Mainz, Mainz, Germany; ^3^ Department of Pharmacology, University Medical Center of the Johannes Gutenberg University Mainz, Mainz, Germany; ^4^ Incyte Biosciences International Sàrl, Morges, Switzerland; ^5^ Incyte Research Institute, Incyte Corporation, Wilmington, DE, United States; ^6^ Transfusion Center, University Medical Center of the Johannes Gutenberg University Mainz, Mainz, Germany; ^7^ Department of Dermatology, University Medical Center of the Johannes Gutenberg University Mainz, Mainz, Germany; ^8^ Institute of Immunology, University Medical Center of the Johannes Gutenberg University Mainz, Mainz, Germany; ^9^ Research Center of Immune Therapy, University Medical Center of the Johannes Gutenberg University Mainz, Mainz, Germany

**Keywords:** T cell, activation, human, granulocyte, arginase 1, neutrophils

## Abstract

Myeloid cell arginase-mediated arginine depletion with consecutive inhibition of T cell functions is a key component of tumor immune escape. Both, granulocytic myeloid-derived suppressor cells (G-MDSC) and conventional mature human polymorphonuclear neutrophil granulocytes (PMN) express high levels of arginase 1 and can act as suppressor cells of adaptive anti-cancer immunity. Here we demonstrate that pharmacological inhibition of PMN-derived arginase 1 not only prevents the suppression of T cell functions but rather leads to a strong hyperactivation of T cells. Human PMN were incubated in cell culture medium in the absence or presence of an arginase inhibitor. T cells from healthy donors were then activated either polyclonally or in an antigen-specific manner in the supernatants of the PMN cultures at different PMN-T cell ratios. T cell proliferation was completely suppressed in these supernatants in the absence of an arginase inhibitor. Arginase inhibition led to a strong hyperinduction of T cell proliferation, which exceeded control activation conditions up to 25-fold. The hyperinduction was correlated with higher PMN-T cell ratios and was only apparent when PMN arginase activity was blocked sufficiently. The T cell stimulatory factor was liberated very early by PMN and was present in the < 3 kDa fraction of the PMN supernatants. Increased T cell production of specific proinflammatory cytokines by PMN supernatant in the presence of arginase inhibitor was apparent. Upon arginase inhibition, downregulation of important T cell membrane activation and costimulation proteins was completely prevented or *de novo* induction accelerated. Antigen-specific T cell cytotoxicity against tumor cells was enhanced by PMN supernatant itself and could be further increased by PMN arginase blockade. Finally, we analyzed anergic T cells from multiple myeloma patients and noticed a complete reversal of anergy and the induction of strong proliferation upon T cell activation in PMN supernatants by arginase inhibition. In summary, we discovered a potent PMN-mediated hyperactivation of human T cells, which is apparent only when PMN arginase-mediated arginine depletion is concurrently inhibited. Our findings are clearly relevant for the analysis and prevention of human tumor immune escape in conjunction with the application of arginase inhibitors already being developed clinically.

## Introduction

An immunosuppressive microenvironment is present in a wide variety of different cancer entities (1). Several cell types and mechanisms are involved in the generation of this pro-tumoral micromilieu, which as a consequence fosters cancer cell proliferation by inhibition of endogenous anti-tumor mechanisms. One key component of this induced tumor immune escape is the expression of the enzyme arginase by infiltrating myeloid cells. Arginase hydrolyzes extracellular arginine to ornithine and urea. T cells require a sufficient amount of extracellular arginine to proliferate and function (2–4). Arginine depletion impairs formation of the immunological synapse causing inhibition of signal transduction through the T cell receptor (5) and arrests T cells in the G_0_–G_1_ phase of the cell cycle (6) whereas supplementation of arginine favors oxidative phosphorylation and increases T cell viability and anti-tumoral activity (7).

Although the mechanism and consequences of arginase-mediated arginine depletion on T cells have been quite uniformly reported, this does not hold true for the nature of the cancer-associated arginase-expressing myeloid cells. While arginase 1 expression can be induced in all murine myeloid cell types, in humans it is mainly found constitutively expressed in granulocytic cells (8, 9). Among these, so-called granulocytic myeloid-derived suppressor cells (G-MDSC) are generally discriminated from normal physiological polymorphonuclear neutrophil granulocytes (PMN) (10). G-MDSC are defined functionally: if their depletion or inhibition of their effector pathways increases anti-tumor immunity in cancer models *in vivo* or if *in vitro* assays demonstrate their suppression of T cell activation (10). Human G-MDSC are still not defined by specific surface markers and it is unclear if they represent a unique granulocytic cellular subtype or rather an activation state of granulocytic cells and if so, whether this activation state is permanent or temporary and being modulated by external inflammatory or tumor-derived factors (11). Most importantly, their relationship to conventional mature PMN, which are the most prevalent myeloid cell type in humans, is unresolved.

These conventional PMN have long been considered only as the short-lived first line defenders against invading pathogens, but recent studies have revealed additional important roles in regulating adaptive immunity (12–14). In addition to their constitutive expression of immunosuppressive arginase, activated PMN produce e.g. reactive oxygen species (ROS), anti-inflammatory cytokines or express cell membrane-bound or released serine proteases, which all inhibit T cell activation (15, 16). In contrast to this, PMN also have immunostimulatory effects. They can secrete proinflammatory cytokines, directly act as antigen presenting cells toward T cells or foster antigen presentation indirectly *via* dendritic cells (DCs) (14, 17). Such a dichotomous potential of PMN has also been described in mice and men for so-called tumor-associated neutrophils (TAN) in different cancer entities: they can behave both pro- or anti-tumoral (18). *In vivo*, T cells in a tumor microenvironment are exposed to infiltrating granulocytic cells, which either secrete factors or liberate intracellular constituents in the process of apoptotic or necrotic cell death.

We therefore wanted to address this complexity in a simplified *in vitro* scenario that would enable us to study the impact of PMN-liberated arginase 1 in conjunction with possible PMN-derived stimulatory factors on T cell activation. We generated supernatants (SN) of PMN from healthy blood donors and analyzed human T cell activation in the context of these PMN-SN. We show that T cell proliferation and other specific features of T cell activation are completely inhibited by high levels of PMN-derived arginase. Unexpectedly, inhibition of arginase during PMN-SN generation was not only able to reconstitute T cell activation, but rather led to a strong hyperstimulation of T cell proliferation, cytokine secretion and cytotoxicity. Our data therefore unravel potent opposing features of normal peripheral blood PMN: they can both, induce a profound immunosuppression and strongly enhance adaptive immunity.

## Materials and Methods

### Human Subjects

This study was approved by Rhineland-Palatinate Medical Association Ethics Committee. Blood donors and Multiple Myeloma (MM) patients gave written informed consent in accordance with the Declaration of Helsinki.

### Reagents

If not otherwise stated, chemicals were purchased from Sigma-Aldrich (St. Louis, MO, USA). Arginase inhibitors: nor-NOHA was purchased from Bachem (Weil am Rhein, Germany) and INCB001158 was provided by Incyte (Wilmington, DE, USA) and Calithera (San Francisco, CA, USA). For cell culture studies arginine-free RPMI 1640 medium (Sigma Aldrich, St Louis, MO, USA) was supplemented with 10% dialyzed FCS, 2 mM L-glutamine, 400 µM L-leucine, 220 µM L-lysine, 150 µM L-arginine, 20 µM MnCl_2_, 100 U/ml penicillin and 0.1 mg/ml streptomycin. [^3^H]thymidine was purchased from Perkin Elmer (Waltham, MA, USA).

### Isolation of T Cells and PMN From Peripheral Blood

T cells and PMN were purified from peripheral blood of either healthy human donors or of MM patients. Ficoll density gradient centrifugation was performed as described before (19). After 15 min of centrifugation (700 *g*), PBMC were harvested from the interphase and CD3^+^ T cells were isolated with the EasySep™ Human T cell Enrichment Kit. CD4^+^ T cells were isolated from PBMC with the EasySep™ Human CD4^+^ T cell Isolation Kit and CD8^+^ T cells were isolated from PBMC with the EasySep™ Human CD8^+^ T cell Isolation Kit (all T cell kits from Stemcell Technologies, Vancouver, Canada). The purity of T cell preparations, which were used for the experiments, was always >95% (mean: 98.3 ± 0.9%). The pellet containing PMN and erythrocytes was resuspended in Dulbecco´s phosphate buffered saline with 1 mM EDTA (PBS/EDTA) and mixed at a ratio of 1:1 with 3% dextran/PBS. After 20 min of erythrocyte-sedimentation the PMN-rich supernatant was harvested and the remaining erythrocytes were subjected to hypotonic lysis (174 mM NH_4_Cl, 10 mM KHCO_3_, 0.1 mM EDTA, pH 7.3) for 15 min on ice. After washing, purity and viability of isolated T cells and PMN were checked by flow cytometry. The purity of PMN cell preparations, which were used for the experiments, was always >95% (mean: 98.6 ± 1.3%). T cells were cultured in RPMI 1640, supplemented with 10% AB serum, 2.5% HEPES, 2 mM L-glutamine, 100 U/ml penicillin and 0.1 mg/ml streptomycin.

### Cell Lines and Culture

Myeloma cell lines FD50 (generated in our laboratory as novel immortalized cell line from a patient with primary plasma cell leukemia), U266 and NCI-H929 (both provided by Prof. Dr. M. Hundemer, University Hospital Heidelberg, Germany) as well as cell lines K562-A2.1 (provided by Prof. Dr. T. Wölfel, University Medicine Mainz, Germany) and SAOS-2 (ATCC^®^ HTB-85™) were cultured in RPMI 1640, supplemented with 10% FCS, 2 mM L-glutamine, 100 U/ml penicillin and 0.1 mg/ml streptomycin.

### T Cell Culture in PMN-Pre-Conditioned Medium

Isolated PMN were cultured for 72 h in RPMI 1640 medium at different PMN concentrations, calculated in such a way that the resulting PMN supernatants (PMN-SNs) could then be used directly at defined PMN to T cell ratios, as indicated in the respective experiments. PMN concentrations for the generation of PMN-SN started at 5 × 10^4^ PMN/200 µl for a PMN:T ratio of 1:1 and PMN concentrations were increased accordingly (2,5 × 10^5^ PMN/200 µl for a PMN:T ratio of 5:1; 5 × 10^5^ PMN/200 µl for a PMN:T ratio of 10:1; 1 × 10^6^ PMN/200 µl for a PMN:T ratio of 20:1). PMN incubation was performed either with or without the addition of the arginase inhibitors nor-NOHA (final concentration: 1 mM) or INCB001158 (final concentration 100 µM). PMN pre-conditioned medium was harvested and centrifuged for 15 min (956 *g*). After centrifugation, T cells (5 × 10^4^ T cells/200 µl) were cultured under varying activation conditions in the PMN-SNs for 48 h.

### Proliferation Assays

T cell proliferation was assessed by the incorporation of [^3^H]thymidine as described before (20). Additionally, T cells were loaded with 25 μM 5-(and 6)-carboxyfluorescein diacetate succinimidyl ester (CFSE, Invitrogen, Carlsbad, CA, USA, as specified before (20), and cultured in PMN-pre-conditioned medium for 96 h. T cell proliferation was quantified by analyzing CFSE dilution by flow cytometry (BD FACSCanto™ II, BD Biosciences, San Jose, CA, USA).

### Flow Cytometry

The following anti-human antigen antibodies were used for flow cytometry: CD3-FITC (clone UCHT1), CD3-APC (clone UCHT1), CD4-FITC (clone RPA-T4), CD8-APC (clone RPA-T8), CD16-PE (clone 3G8), CD25-PE (clone BC96), CD28-FITC (clone CD28.2), CD66b-FITC (clone G10F5), CD69-PE (clone FN50), CD279-FITC (clone MIH4), Vβ-3-PE (clone KJ25) from BD Biosciences (San Jose, CA, USA); CD152-APC (clone L3D10) from BioLegend (San Diego, CA, USA); LAG-3-PerCP (#FAB2319C) and TIM-3-AlexaFluor488 (clone 344823) from R&D Systems (Minneapolis, MN, USA); CD57-PE-Cy7 (clone TB01) from eBioscience (Thermo Fisher Scientific, Waltham, MA, USA), TIGIT-PE (clone MBSA43) from Invitrogen (Thermo Fisher Scientific, Waltham, MA, USA). For flow cytometry analysis, 0.2 × 10^6^ cells were pelleted, 1 µl of the antibody was added and the tube was vortexed shortly. After 15 min of incubation in the dark, cells were washed with 1 ml PBS and resuspended in 200 µl PBS/1% PFA. Analysis was done at BD FACS Canto™ II (BD Biosciences, San Jose, CA, USA).

### IFN-*γ* ELISA and Cytokine Bead Array of Human T Cell Supernatants

For IFN-*γ* detection, T cell culture supernatants were harvested after 48 h of incubation and IFN-*γ* was determined using the OptEIA Human IFN-*γ* ELISA Set (Becton Dickinson, Heidelberg, Germany) according to the manufacturer´s instructions. All other cytokines were analyzed in culture supernatants after 48 h of incubation by cytometric bead array using the human CBA flex sets according to the manufacturer´s instructions (BD Biosciences, San Jose, CA, USA).

### Arginase Enzymatic Assay

Arginase activity was measured in supernatants after PMN pre-culture as previously described (8) with slight modifications. Briefly, protein concentrations in PMN supernatants were determined with *DC*™ Protein Reagents Package (Bio-Rad, Hercules, CA, USA) according to the manufacturer´s instructions. For each condition, an aliquot of the supernatant that contained 3.3 µg total protein was diluted in a total of 100 µl PBS and 20 µl of 10 mM MnCl_2_ was added. The enzyme was activated by heating for 10 min at 56°C. Arginine hydrolysis and measurement of urea concentration were performed exactly as previously described (8). One unit of enzyme activity is defined as the amount of enzyme that catalyzes the formation of 1 µmol urea per minute.

### Quantification of L-Arginine Concentration

To 50 µl cell culture supernatants, 4 nmol N^G^-monomethyl-L-arginine (L-NMMA) were added as internal standard, then supplemented with 0.9 ml PBS (pH 6.9) and applied to an Oasis MCX ion exchange column (Waters, Eschborn, Germany). The column was washed with 1 ml each, 0.1 N HCl and methanol, and subsequently cationic amino acids (CAA) were eluted with 1 ml methanol:water: 25% NH_3_ (5:4:1), vacuum dried and resuspended in 0.2 ml sodium borate buffer (0.5 mol/l, pH 9.6). L-arginine levels were determined in cell culture supernatants by High Performance Liquid Chromatography (HPLC) using precolumn derivation, exactly as described before (20, 21).

### Tumor Killing Assay

Gillies crystal violet staining is a spectrophotometric method for quantitative cell count determination of adherent cells (22) which can also be used to quantify cellular cytotoxicity. Human T cells were retrovirally transduced with a single-chain T cell receptor (scTCR) with specificity against p53(264-272) as previously described (23). TCR-modified T cells were cultivated and expanded by weekly restimulation with irradiated p53(264-272)-loaded K562-A2.1 cells. For the killing assay, SAOS-2 cells were seeded as follows and cultured overnight: 0.1 × 10^6^ cells per well in a 24-well plate with the addition of 20 ng/ml IFN-*γ* per well (to upregulate HLA-A2 expression). SAOS-2 cells were loaded with 0.1 ng p53(264-272) peptide for 3 h, then medium was discarded and the cells were washed once with PBS. SAOS-2 cells were cultured for 3 h either with transduced T cells (0.6 × 10^6^ T cells in 500 µl per well in a 24-well plate; T cells had been cultured for 48 h in the various activation conditions) or in PMN-SN. SAOS-2 cells were washed, fixed with 4% PFA and remaining cells stained with 0.05% crystal violet and lysed with 5% SDS solution. Absorption was measured at 570 nm.

### Size-Exclusion Ultra-Filtration

PMN-SN was centrifuged through an Amicon^®^ Ultra 10 kDa molecular weight cut-off (MWCO) filter (#UFC901024, Merck Chemicals, Darmstadt, Germany) for 30 min (1,300 *g*). The retentate was diluted to the original volume with RPMI 1640 medium (= PMN-SN > 10 kDa). The flow through was further fractionated by ultrafiltration through an Amicon^®^ Ultra filtration device with a 3 kDa MWCO (#UFC900324, Merck Chemicals) for 30 min (1,300 *g*). The retentate was diluted to the original volume with RPMI 1640 medium (= PMN-SN < 10 kDa and > 3 kDa) and the flow through contained PMN-SN < 3 kDa. Dialyzed FCS (10% v/v) was added to all PMN-SN fractions to replace the FCS-loss due to the first cut-off filtration.

### Statistical Analysis

Statistical analyses were performed with GraphPad Prism software 6. Results are expressed as mean ± SD. Statistical differences were calculated using one-way ANOVA, followed by Tukey *post-hoc* test. The levels of significance were specified as ***p < 0.001, **p < 0.01 and *p < 0.05.

## Results

### Supernatant of Human PMN hyperactivates T Cell Proliferation in the Presence of an Arginase Inhibitor

**Figure 1:**
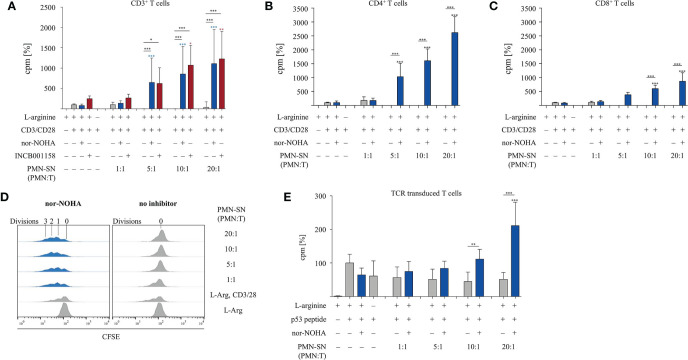
T cell proliferation is strongly enhanced by polymorphonuclear neutrophil granulocytes supernatants (PMN-SN) in the presence of arginase inhibition. **(A–C)** Primary human T cells (bulk CD3^+^ or sorted CD4^+^ or CD8^+^) and PMN were isolated from whole blood of healthy donors. PMN were pre-incubated in cell culture medium for 72 h in the presence or absence of the arginase inhibitor nor-NOHA (1 mM) or INCB001185 (100 µM). T cells were then stimulated for 48 h with anti-CD3/anti-CD28-tagged beads in the PMN-SN. T cell proliferation was determined by [^3^H]thymidine incorporation over 16 h and values of stimulated cells in the presence of arginine were set as 100%. **(A)** CD3^+^ T cells from n=31 individual experiments (n=5 for INCB001185) with T cells from different blood donors, **(B)** CD4^+^ T cells (n=4) and **(C)** CD8^+^ T cells (n=4). **(D)** Human PBMC and PMN were isolated from whole blood of healthy donors. T cells were retrovirally transduced with a HLA-A2 restricted p53(264-272)-specific T cell receptor and expanded by weekly restimulation with anti-CD3/anti-CD28-tagged beads and later *via* p53 peptide-loaded K562-A2.1 cells. PMN-SN was generated as described in **(A–C)**. T cells were then stimulated with p53 peptide-loaded irradiated K562-A2.1 cells in PMN-SN for 48 h. T cell proliferation (n=3) was analyzed as in **(A–C)**. **(E)** Primary human CD3^+^ T cells were activated in PMN-SN for 48 h as described in **(A)**. T cell proliferation was determined by carboxyfluorescein succinimidyl ester (CFSE) staining (one representative experiment of n=7 is shown). Unless otherwise stated, statistical analysis refers to the control conditions of activated T cells in the presence of L-arginine and nor-NOHA. Statistical calculations were performed with one-way ANOVA and Tukey´s *post hoc* test (***p < 0.001, **p < 0.01, *p < 0.05).

T cells and PMN were isolated from the peripheral blood of healthy donors. PMN were incubated at different concentrations (0.25 × 10^6^ – 5.0 × 10^6^ PMN/ml) for 72 h in normal cell culture medium in the absence or presence of the arginase inhibitors nor-NOHA (1 mM) or INCB001158 (100 µM), an arginase inhibitor that was designed for blocking the activity of the extracelluar enzyme and is currently in clinical studies for treating various tumor types. Cell-free supernatants (SN) of these PMN incubation cultures were then harvested (PMN-SN). T cells were then activated by anti-CD3/anti-CD28 tagged beads in the different PMN-SNs for 48 h at different calculated PMN:T cell ratios. T cell proliferation was determined by the incorporation of [^3^H]thymidine over further 16 h. To compensate for interindividual differences in absolute cpm values, the proliferation results were summarized and normalized to control activated T cells (set as 100%, as explained in the individual figure legends). First, arginine-dependence of CD3^+^ T cell activation was verified: while control CD3^+^ T cells proliferated in cell culture medium containing 150 µM L-arginine (4,095 ± 2,416 cpm; n=31 individual experiments with blood from different donors), there was no relevant proliferation detectable in arginine-free cell culture medium (**Figure 1A**). No negative impact on T cell proliferation was detectable when T cells were cultured in a PMN:T ratio of 1:1. When higher PMN numbers were used to generate the PMN-SN, a complete inhibition of T cell proliferation was noted, starting from a PMN:T ratio of 5:1 (**Figure 1A**). Most interestingly, this inhibition of T cell proliferation was not only prevented by the arginase inhibitors, but T cell proliferation was strongly hyperactivated compared to the arginine-containing control conditions without PMN-SN. This hyperactivation of T cell proliferation was positively correlated with increasing PMN density used for the generation of the PMN-SN, achieving a more than 10-fold increase in proliferation at a 20:1 PMN:T ratio (nor-NOHA: 33,875 ± 18,731 cpm; INCB001158: 40,149 ± 26,471 cpm; **Figure 1A**). This T cell hyperstimulation was detectable both in purified CD4^+^ T cells (**Figure 1B**) and in purified CD8^+^ T cells (**Figure 1C**). Comparing both T cell subpopulations, the hyperactivating effect was roughly two times higher in CD4^+^ T cells compared to CD8^+^ T cells. Proliferation of CD4^+^ T cells incubated in PMN-SN at a PMN:T ratio of 20:1 increased roughly 25 times (2,618 ± 558%; n=4) compared to the control activation conditions (150 µM L-arginine and 1 mM nor-NOHA; **Figure 1B**), whereas CD8^+^ T cell proliferation at the same conditions showed a roughly 10-fold increase (872 ± 271%, n=3) in proliferation compared to the respective controls (**Figure 1C**). Hyperactivation was only seen in stimulated T cells, while proliferation could not at all be induced in resting, non-activated T cells by PMN-SN + arginase inhibition (data not shown). We next analyzed this novel hyperactivating effect on the single T cell level by CFSE assay. Therefore, CD3^+^ T cells were labeled with CFSE and activated with anti-CD3/anti-CD28-tagged beads in PMN-SN at different PMN:T ratios for 96 h. Proliferation was analyzed by flow cytometry with loss of CFSE fluorescence correlating to sequential rounds of cell divisions (**Figure 1D**). Again, induced T cell proliferation was inhibited by PMN-SN: the number of undivided T cells increased with higher PMN density, when arginase was not inhibited in PMN-SN. However, T cell proliferation was hyperactivated when the arginase inhibitor nor-NOHA had been added during PMN-SN generation. The CFSE assay demonstrated that this hyperactivation was really driven by productive rounds of T cell division, leading to cell cycling of the majority of T cells and increasing the absolute numbers of cell cycle rounds in the entire population: the number of divided T cells increased within the first, second and third generation in a PMN concentration-dependent manner (**Figure 1D**). Finally, we analyzed potential T cell hyperactivation in an antigen-specific T cell population. T cells were retrovirally transduced with a HLA-A2 restricted, p53(264-272) peptide-specific TCR and expanded by weekly restimulation with anti-CD3/anti-CD28-tagged beads. These T cells were then activated in PMN-SN for 48 h antigen-specifically by using p53(264-272) peptide pre-pulsed, irradiated K562-A2.1 cells. T cell activation was clearly p53 peptide-dependent, but only moderately suppressed by arginine deficiency or by activation in PMN-SN without nor-NOHA. Most importantly however, an antigen-specific hyperinduction of T cell proliferation was again seen when arginase was concurrently blocked in PMN-SN by nor-NOHA (**Figure 1E**).

### Very Early Immunostimulatory Effect of PMN Precedes Arginase-Driven Inhibition of T Cell Proliferation

**Figure 2:**
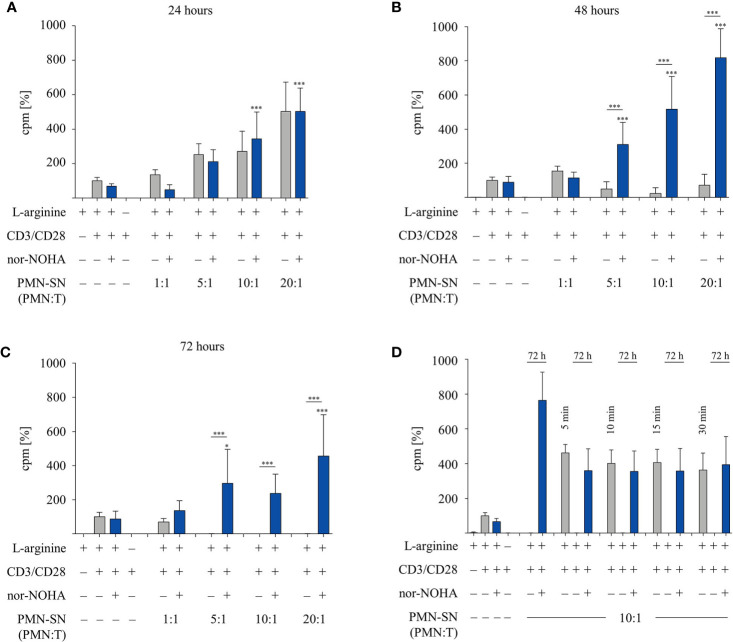
A pronounced T cell immunostimulatory effect is detected very early in polymorphonuclear neutrophil granulocytes supernatants (PMN-SN), while arginase-mediated inhibition of T cell proliferation needs much longer PMN-SN incubation time. Human T cells and PMN were isolated from whole blood of healthy donors. PMN were incubated in cell culture medium for **(A)** 24 h, **(B)** 48 h, or **(C)** 72 h in the presence or absence of the arginase inhibitor nor-NOHA (1 mM; n=4 experiments). **(D)** PMN were incubated in cell culture medium for only 5 min, 10 min, 15 min, or 30 min. Supernatants from these short incubations were collected and PMN were incubated for another 72 h in fresh medium in the presence or absence of nor-NOHA (1 mM; n=3). **(A–D)** T cells were stimulated with anti-CD3/anti-CD28-tagged beads and cultured in PMN-SN for 48 h. T cell proliferation was determined by [^3^H]thymidine incorporation over 16 h and values of control stimulated cells in the presence of arginine were set as 100%. Unless otherwise stated, statistical analysis refers to the control conditions of activated T cells in the presence of L-arginine and nor-NOHA. Statistical calculations were performed with one-way ANOVA and Tukey´s *post hoc* test (***p < 0.001, *p < 0.05).

In order to investigate the two opposite, inhibitory versus stimulatory, effects of PMN on T cells in more detail, we tested different PMN incubation times in further experiments. PMN were incubated for 24 h, 48 h, and 72 h (± nor-NOHA) to generate PMN-SN (**Figures 2A–C**). T cell proliferation was not inhibited in any 24 h PMN-SN containing condition: while again a pronounced hyperactivation of T cell proliferation was induced and increased with PMN density in the PMN-SN, this T cell activation was completely independent of arginase inhibition (**Figure 2A**). Upon 48 h of PMN incubation for the generation of the PMN-SN, the T cell activation phenotype was completely reversed in the absence of arginase inhibitor nor-NOHA: here a pronounced inhibition of T cell proliferation by PMN-SN was now detectable (**Figure 2B**) and a complete shutdown of T cell proliferation was seen when 72 h PMN-SN was used (**Figure 2C**, comparable to **Figure 1A**). Adding the arginase inhibitor not only restored, but strongly activated T cell proliferation when 48 h PMN-SN (**Figure 2B**) or 72 h PMN-SN (**Figure 2C**) were used. Since the hyperactivating factor(s) was/were already present in 24 h PMN-SN, we next focused on much shorter incubation times for the generation of the PMN-SN. Isolated PMN were pre-incubated at a PMN:T ratio of 10:1 for only 5 min, 10 min, 15 min, and 30 min in cell culture medium containing 150 µM L-arginine without nor-NOHA supplementation. After these different incubation times, PMN-SNs were collected and remaining PMN were incubated in fresh cell culture medium for 72 h with or without 1 mM nor-NOHA. T cells were then stimulated in all the different short-term as well as the respective 72 h PMN-SNs for 48 h. PMN incubation for just 5 min led to a PMN-SN that was fostering T cell hyperactivation, reaching 462 ± 50% (n=3) of control T cell proliferation and this was recapitulated with the 10 min – 30 min PMN-SNs (**Figure 2D**). Hyperactivation of T cell proliferation was also seen when the respective 72 h PMN-SN was used for T cell stimulation, but only in the presence of the arginase inhibitor nor-NOHA, while T cell proliferation was completely shut-down in the absence of arginase inhibition. Control 72 h PMN-SN (which was derived as before without harvest of a first short-term PMN-SN and resupplementation of fresh medium) led to a T cell hyperactivation, which quantitatively amounted roughly to the sum of a first short-term PMN-SN and the respective 72 h PMN-SN.

### Arginine Availability Is Necessary for PMN-Driven Hyperactivation of T Cell Proliferation

**Figure 3:**
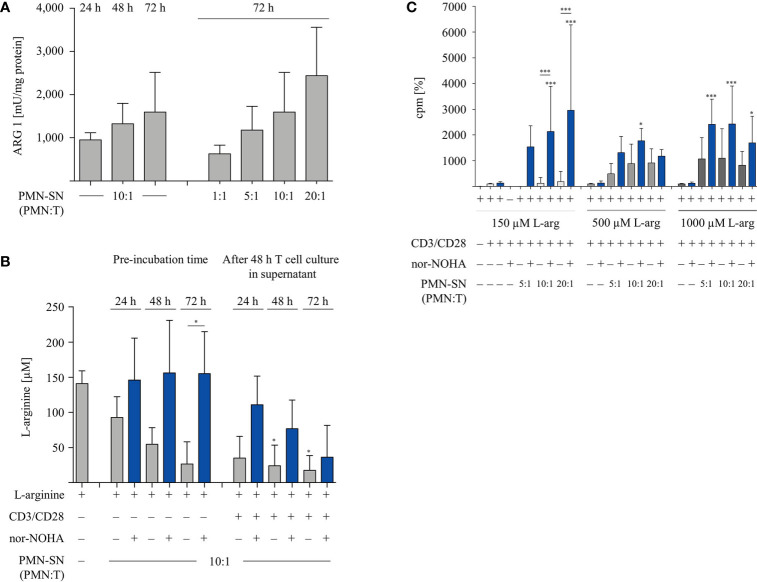
Arginine availability correlates with hyperactivation of T cell proliferation. Human T cells and polymorphonuclear neutrophil granulocytes (PMN) were isolated from whole blood of healthy donors. PMN were incubated in cell culture medium for 24 h, 48 h, or 72 h in the presence or absence of 1 mM nor-NOHA. **(A)** Arginase activity was determined in PMN-supernatants (SN) at different PMN concentrations corresponding to the indicated PMN:T ratios in the following T cell activation assays (n=4). **(B)** T cells were stimulated with anti-CD3/anti-CD28-tagged beads for 48 h in the different PMN-SN. L-arginine concentrations were quantified by high performance liquid chromatography in the PMN-SN before and after 48 h of T cell activation. Arginine values of normal cell culture medium were set as 100% (n=3 independent experiments). **(C)** PMN were incubated in cell culture medium containing 150 µM, 500 µM, or 1,000 µM L-arginine for 72 h in the presence or absence of 1 mM nor-NOHA. T cells were then stimulated with anti-CD3/anti-CD28-tagged beads in the PMN-SN for 48 h. T cell proliferation was determined by [^3^H]thymidine incorporation over 16 h and values of control stimulated cells in the presence of arginine were set as 100% (n=4 independent experiments). Unless otherwise stated, statistical analysis refers to the control conditions of activated T cells in the presence of L-arginine and nor-NOHA. Statistical calculations were performed with one-way ANOVA and Tukey´s *post hoc* test (***p < 0.001, *p < 0.05).

Since PMN-SN induced T cell hyperactivation was only apparent in the context of arginase inhibition at extended PMN-SN incubation times (**Figure 2A**), we next analyzed arginase expression and arginine availability directly. Isolated PMN were pre-incubated in culture medium with or without nor-NOHA supplementation for 24 h, 48 h, and 72 h. Subsequently, PMN-SN were collected for arginase activity determination by biochemical assay and for arginine quantification *via* high performance liquid chromatography (HPLC). Additionally, anti-CD3/anti-CD28-stimulated T cells were cultured for 48 h in the supernatants (at a calculated PMN-T cell ratio of 10:1) and arginine levels in these supernatants were also quantified with HPLC. Arginase activity in the PMN-SN was already detectable after 24 h (mean: 949 ± 165 mU/mg protein; n=3), and increased further at 48 h (1,321 ± 477 mU/mg protein; n=3) and 72 h (1,596 ± 919 mU/mg protein; n=3; **Figure 3A**). Arginine concentrations in the PMN supernatants without arginase inhibitor decreased over time, which was efficiently prevented by addition of 1 mM nor-NOHA (**Figure 3B**). In 72 h PMN-SN, arginine concentrations had dropped from 141.3 ± 18.1 µM at start to 26.8 ± 31.4 µM, which is below the level required for maintaining T cell proliferation *in vitro*, without arginase inhibition but remained at 155.3 ± 59.9 µM (n=3) in the presence of nor-NOHA. When T cells were incubated for 48 h in these different supernatants, arginine concentrations were further decreasing, which is likely arginase-mediated (grey bars, in parallel: no T cell proliferation detectable) or due to arginine uptake and metabolism of strongly proliferating T cells (conditions with nor-NOHA, blue bars). Since these experiments demonstrated a clear correlation of arginine availability and the potential of PMN-SN to boost T cell proliferation, we next examined the impact of supplemented higher arginine concentrations on PMN-driven hyperactivation and, *vice versa*, PMN arginase-mediated inhibition of T cell proliferation (**Figure 3C**). PMN were incubated for 72 h at different concentrations in cell culture media, supplemented with 150 µM, 500 µM, or 1,000 µM arginine, at each concentration either with or without the addition of 1 mM nor-NOHA. T cells were then stimulated with anti-CD3/anti-CD28-tagged beads in the different PMN-SN for 48 h. Hyperactivation of T cell proliferation was induced in all tested PMN-SN conditions when arginase was inhibited by nor-NOHA. This hyperactivation was apparent at physiological arginine concentrations (150 µM) and was not further boosted by increasing arginine exogenously to supraphysiological concentrations. On the other hand, this surplus of arginine prevented the PMN-SN mediated shut-down of T cell proliferation and rather enabled T cell hyperactivation, as well (**Figure 3C**). These data clearly confirm that there is an arginine-dependent immunostimulatory effect of PMN-derived factor(s) on T cells.

### PMN-Mediated Hyperactivation of T Cell Cytokine Secretion

**Figure 4:**
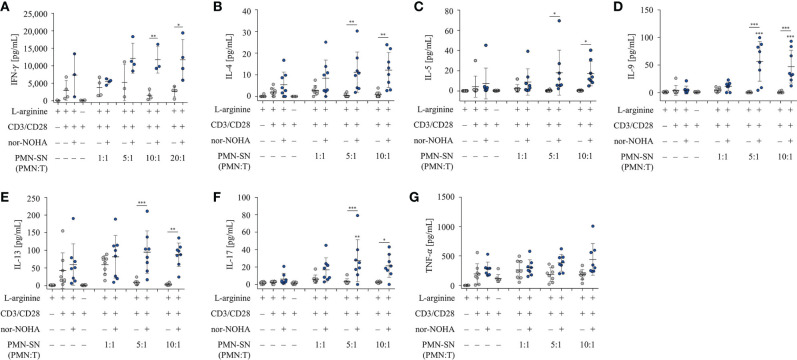
T cell cytokine secretion is hyperactivated by polymorphonuclear neutrophil granulocytes supernatants (PMN-SN) in the presence of arginase inhibition. Human T cells and PMN were isolated from whole blood of healthy donors. PMN were pre-incubated for 72 h in the presence or absence of the arginase inhibitor nor-NOHA (1 mM). T cells were stimulated with anti-CD3/anti-CD28-tagged beads in the PMN-SN for 48 h. **(A)** IFN-γ concentrations were quantified in the supernatants of these T cell activation cultures by ELISA and values of control stimulated cells in the presence of arginine were set as 100% (n=4 independent experiments). **(B–G)** Cytokines in T cell culture supernatants were analyzed by cytometric bead array (n=8 independent experiments). Unless otherwise stated, statistical analysis refers to the control conditions of activated T cells in the presence of L-arginine and nor-NOHA. Statistical calculations were performed with one-way ANOVA and Tukey´s *post hoc* test (***p < 0.001, **p < 0.01, *p < 0.05).

Next, we examined the secretion of IFN-γ from hyperactivated T cells. T cells were activated according to the standard hyperactivating conditions in PMN-SN for 48 h. Supernatants of these T cell activation assays were collected and analyzed by IFN-γ ELISA (**Figure 4A**). An arginine-dependent IFN-γ secretion was detected in activated T cells, as has been published previously (24). IFN-γ secretion was further increased in hyperactivated T cells cultured in PMN-SN with PMN:T ratios greater than 5:1, compared to conventionally activated T cells (**Figure 4A**). At higher PMN concentrations in PMN-SN in the absence of arginase inhibitor a suppression of IFN-γ secretion was detectable, reaching levels of conventionally anti-CD3/anti-CD28-activated T cells. Additionally, we investigated the secretion of further cytokines from hyperactivated T cells. T cell culture supernatants were collected after 48 h standard PMN-SN culture conditions and analyzed by Cytokine Bead Array (CBA) (**Figures 4B–G**). An arginine-dependent secretion was detected for the cytokines IL-4 (**Figure 4B**), IL-5 (**Figure 4C**), IL-9 (**Figure 4D**), IL-13 (**Figure 4E**), and IL-17 (**Figure 4F**). Among these cytokines, hyperactivated T cells secreted significantly higher levels of IL-9 and IL-17 compared to conventionally activated T cells. IL-9 secretion was significantly higher in T cells cultured in PMN-SN with a PMN:T ratio of 5:1 (56.6 ± 53.9 pg/ml, n=8) and 10:1 (47.1 ± 29.8 pg/ml, n=8) compared to conventionally activated T cells (4.7 ± 7.1 pg/ml, n=8, **Figure 4D**). Hyperactivated T cells showed a higher IL-17 secretion at a PMN:T ratio of 5:1 (27.5 ± 23.5 pg/ml, n=8) than conventionally activated T cells (6.2 ± 6.6 pg/ml, n=8, **Figure 4F**). For the type 2 T helper cell (Th2) cytokines IL-4, IL-5, and IL-13 there was only a trend, but no significant induction of cytokine secretion in hyperactivated T cells compared to conventionally activated T cells. In contrast, secretion of TNF-α was not affected by arginine availability or T cell hyperactivation, but was rather stable in all conditions (**Figure 4G**). Comparable results were seen when the alternative arginase inhibitor INCB001158 was used in cytokine bead array experiments (**Supplementary Figure 1**).

### Hyperactivated T Cells Have an Increased and Faster (Re-)Expression of T Cell Activation Markers, While Suppressive Checkpoint Inhibitor Molecules Are Not Induced

**Figure 5:**
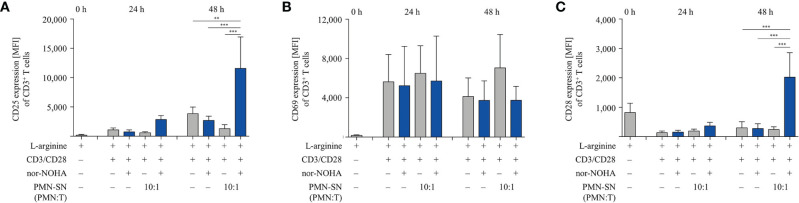
Hyperactivated T cells have an earlier and/or increased expression of CD25 and CD28. Human T cells and polymorphonuclear neutrophil granulocytes (PMN) were isolated from whole blood of healthy donors. PMN were incubated for 72 h in the presence or absence of nor-NOHA (1 mM). T cells were stimulated with anti-CD3/anti-CD28-tagged beads in the PMN-SN or in medium with or without the addition of 1 mM nor-NOHA for 24 h and 48 h. T cells were then harvested and stained with **(A)** anti-CD25, **(B)** anti-CD69, or **(C)** anti-CD28 antibodies and analyzed by flow cytometry. A summary of expression (MFI of CD3^+^ cells) of n=3 independent experiments with blood from different donors is demonstrated. Statistical calculations were performed with one-way ANOVA and Tukey´s *post hoc* test (***p < 0.001, **p < 0.01).

**Figure 6:**
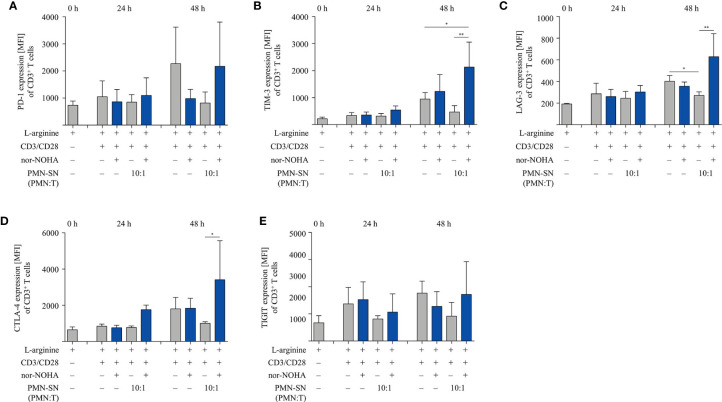
Expression of suppressive checkpoint inhibitor molecules is not enhanced by T cell activation under hyperstimulatory conditions. Human T cells and polymorphonuclear neutrophil granulocytes (PMN) were isolated from whole blood of healthy donors. PMN were incubated for 72 h in the presence or absence of nor-NOHA (1 mM). T cells were stimulated with anti-CD3/anti-CD28-tagged beads in the PMN-SN or in medium with or without the addition of 1 mM nor-NOHA for 24 h and 48 h. T cells were then harvested and stained with **(A)** anti-PD-1, **(B)** anti-TIM-3, **(C)** anti-LAG-3, **(D)** anti-CTLA-4, or **(E)** anti-TIGIT antibodies and analyzed by flow cytometry. A summary of expression (MFI of CD3^+^ cells) of n=3 independent experiments with blood from different donors is demonstrated. Statistical calculations were performed with one-way ANOVA and Tukey´s *post hoc* test (**p < 0.01, *p < 0.05).

We next studied important activation-associated T cell membrane proteins. CD3^+^ T cells were stimulated with anti-CD3/anti-CD28-tagged beads in PMN supernatant (PMN:T ratio 10:1) with and without nor-NOHA for 24 h and 48 h. Subsequently, T cells were stained with antibodies against CD25, CD69 and CD28 and analyzed by flow cytometry (**Figure 5**). The Interleukin-2 receptor α-chain (CD25), an arginine-dependent activation-associated protein (24) was expressed in the majority of T cells (75.7 ± 8.6% of CD3^+^ cells; mean fluorescence intensity, MFI: 2863 ± 667) already after 24 h of activation in nor-NOHA containing PMN-SN, while conventionally activated T cells only expressed CD25 in 40.4 ± 3.3% (MFI: 740 ± 345) of the cell population (**Figure 5A**). At 48 h, these differences in MFI expression levels further increased (MFI in hyperactivated cells: 11,573 ± 5,353 versus MFI in conventionally activated cells: 2,685 ± 737) In PMN-SN without arginase inhibitor, much less activated T cells expressed CD25 at all points of time tested. In contrast to CD25, the activation-inducible expression of the early activation marker CD69 is arginine-independent (24). In our novel hyperactivating assay conditions we also saw an upregulation of CD69, which was independent of the presence of hyperactivating PMN-SN, both in the absence or presence of nor-NOHA. At all points of time and in all assay conditions we measured comparable fractions of CD69-expressing T cells (**Figure 5B**). CD28 is a constitutively expressed surface glycoprotein with important co-stimulatory effects on T cell proliferation and effector functions. Upon anti-CD3/anti-CD28-mediated activation, a downregulation of CD28 expression was detectable on T cells. CD28 was then re-expressed in conventionally activated T cells. Under hyperactivation conditions, T cells started to re-express CD28 already after 24 h, exceeding pre-activation levels after 48 h (**Figure 5C**). In contrast, T cell activation in PMN-SN without arginase inhibition completely prevented re-expression of this important costimulatory molecule. We also investigated effects of hyperactivation on membrane proteins, which are associated with T cell suppression or exhaustion: Programmed cell death-1 (PD-1), cytotoxic T lymphocyte antigen-4 (CTLA-4), T cell immunoglobulin domain and mucin domain-containing protein 3 (TIM-3), T cell immunoreceptor with Ig and ITIM domains (TIGIT) and lymphocyte-activation gene 3 (LAG-3) (25). We noted a continuous increase in activation-induced expression over time for all these surface proteins (**Figures 6A–E**). We did not detect significant differences between hyperactivated (PMN-SN + nor-NOHA) and conventionally activated T cell in terms of time kinetic. Absence of the arginase inhibitor nor-NOHA in PMN-SN-activated T cells led to an inability of the cells to upregulate these surface proteins, reflecting the absence of cellular activation under these profound inhibitory conditions.

### T Cell Cytotoxicity Is Stimulated by PMN-SN and Further Increased by Arginase Inhibition

**Figure 7:**
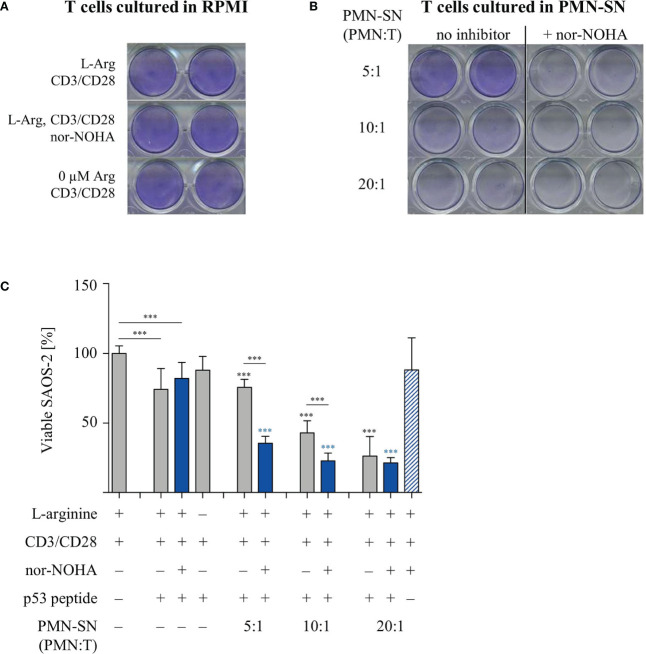
Hyperactivation of T cell cytotoxicity is induced by polymorphonuclear neutrophil granulocytes supernatants (PMN-SN) and further increased by arginase inhibition. Human T cells and PMN were isolated from whole blood of healthy donors. Human T cells were retrovirally transduced with an HLA-A2 restricted p53(264-272)-specific T cell receptor and expanded by weekly restimulation with anti-CD3/anti-CD28-tagged beads and later *via* p53 peptide-loaded K562-A2.1 cells. PMN were incubated in cell culture medium for 72 h in the presence or absence of the arginase inhibitor nor-NOHA (1 mM). T cells were stimulated with anti-CD3/anti-CD28-tagged beads and cultured in the PMN-SN for 48 h. SAOS-2 cells, pulsed with p53(264-272) peptide as indicated, were cultured for 3 h with T cells **(A)** in the absence or **(B)** in the presence of PMN-SN at the indicated conditions. SAOS-2 cells were then stained with crystal violet to detect viable cells. **(C)** Quantification of viable SAOS-2 cells (n=3 independent experiments): OD values of control SAOS-2 cells (see **Supplementary Figure S2**) were set as 100% and OD values of the different experimental conditions were normalized to these controls. Statistical analysis: if not otherwise indicated, black asterisks refer to the control condition of SAOS-2 cells without p53 peptide (leftmost column), while blue asterisks refer to the control condition of SAOS-2 cells pulsed with p53 peptide and incubated with T cells in the absence of PMN-SN (leftmost blue column). Statistical calculations were performed with one-way ANOVA and Tukey´s *post hoc* test (***p < 0.001).

T cell cytotoxicity is a crucial function within the anti-cancer immune response. We therefore analyzed the cytotoxic potential of primary human T cells in the context of PMN-derived factors with or without arginase inhibition. T cells from healthy donors were retrovirally equipped with a HLA-A2-restricted, p53(264-272) peptide-specific TCR and expanded and selected *in vitro*. PMN-SN was generated from another healthy donor with or without the addition of 1 mM nor-NOHA for 72 h and p53-transduced and anti-CD3/anti-CD28 activated T cells were then incubated in the PMN-SN for 48 h. Subsequently, T cells were harvested and co-cultured with p53(264-272) peptide-loaded SAOS-2 cells at an effector:target ratio of 3:1 in normal RPMI cell culture medium (**Figure 7A**) or in the respective PMN-SN (**Figure 7B**). The co-culture was stopped as soon as a cytotoxic effect was observed by visual control (3 h) and cellular SAOS-2 cytotoxicity was quantified by crystal violet assay. Assay conditions were chosen in such a way, that an only moderate cytotoxic effect was seen in control conditions with co-incubation of SAOS-2 cells and p53-specific T cells upon p53 peptide pulsing (74.1 ± 16.9% viability compared to controls without p53 peptide pulsing, set as 100%), so as to clearly highlight potential differences between conventionally activated and hyperactivated T cells. With increasing PMN numbers in PMN-SN a pronounced increase in T cell cytotoxicity became obvious. In contrast to the other T cell functions tested so far, this hyperactivation of T cell cytotoxicity was apparent even without concurrent arginase inhibition in PMN-SN. However, when nor-NOHA was added, the activation of T cell cytotoxicity was even further enhanced. Again, this hyperactivation became more noticeable with increasing PMN density, reducing tumor cell viability to 35.4 ± 5.6% at a PMN:T ratio of 5:1, to 22.8 ± 6.2% at a PMN:T ratio of 10:1 and to 21.3 ± 4.3% at a PMN:T ratio of 20:1 (**Figure 7C**). This killing was still strictly antigen-dependent, since no killing was observed with SAOS-2 cells in the absence of p53 peptide loading (**Figure 7C**, blue striped bar) and SAOS-2 cell viability was unimpaired in all conditions in the absence of antigen-specific T cells (**Supplementary Figure 2**).

### T Cell Anergy of Patients With Multiple Myeloma Is Broken by PMN and Arginase Inhibition While Proliferation of Myeloma Cell Lines Is Not Stimulated

**Figure 8:**
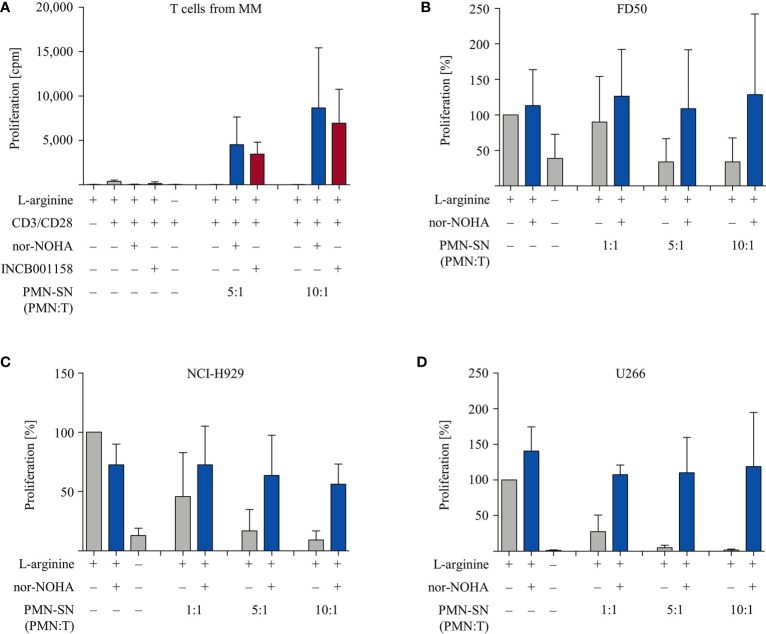
Multiple Myeloma (MM): anergic T cells from MM patients are hyperactivated while proliferation of MM cell lines is not enhanced. **(A)** T cells and polymorphonuclear neutrophil granulocytes (PMN) were isolated from whole blood of MM patients. PMN were incubated in cell culture medium for 72 h in the presence or absence of the arginase inhibitors nor-NOHA (1 mM) or INCB001158 (100 µM), as indicated. T cells were then stimulated with anti-CD3/anti-CD28-tagged beads in the PMN-SN for 48 h. T cell proliferation was determined by [^3^H]thymidine incorporation over 16 h and values of stimulated cells in the presence of arginine were set as 100%. Summary data from n=6 individual MM patients are shown. **(B–D)** Cells from different myeloma cell lines were cultured in PMN-SN [generated as in **(A)**] for 48 h at the indicated conditions. Cancer cell proliferation was then determined by [^3^H]thymidine incorporation over 16 h and values of cells in the presence of arginine (150 µM; without PMN-SN) were set as 100%. **(B)** FD50 cells, **(C)** NCI-H929 cells, and **(D)** U266 cells (results for each cell line from n=3 independent experiments).

Finally, we studied if the PMN-derived factor(s) in the context of arginase inhibition can also hyperactivate functionally impaired T cells from cancer patients. To address this question we collected T cells from the peripheral blood of multiple myeloma (MM) patients. These cells are known to express enhanced exhaustion and senescence markers and to proliferate less upon activation in comparison to T cells from healthy blood donors (26). Our data confirm this, since purified T cells from n=6 different MM patients proliferated much less upon anti-CD3/anti-CD28 mediated activation (377 ± 157 cpm) in comparison with T cells from healthy donors (4,095 ± 2,416 cpm). We subjected these T cells to our novel activation conditions (± patient-derived PMN-SN, ± nor-NOHA or ± INCB001158). The profound T cell anergy was reversed by MM patient-derived PMN-SN, if arginase was inhibited (**Figure 8A**). Again, this hyperactivation was increased with higher PMN density during PMN-SN generation. To study whether myeloma cancer cell proliferation can also be stimulated by PMN-derived factor(s), we analyzed the effects of PMN-SN (± nor-NOHA) on three different myeloma cell lines. The experimental set up was identical to the T cell assays, but now the proliferation of FD50 (**Figure 8B**), NCI-H929 (**Figure 8C**), and U266 (**Figure 8D**) cells was quantified. Proliferation of all three cell lines was inhibited when they were cultured in the absence of arginine or in the presence of PMN-SN. When nor-NOHA was added during PMN-SN generation, this PMN-SN-induced suppression of myeloma cell proliferation was prevented. Importantly, reconstitution of myeloma cell proliferation only reached the level of their proliferation in normal, arginine-containing cell culture medium. Under all tested PMN:T ratios in the presence of nor-NOHA, there was no hyperproliferation of cancer cells (**Figures 8B–D**).

### T Cell Hyperactivation Is Induced by PMN-Derived Factor(s) < 3 kDa, Which Are Quite Heat-Stable

**Figure 9:**
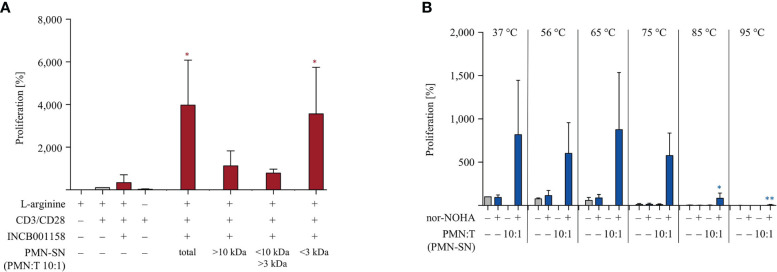
The polymorphonuclear neutrophil granulocytes (PMN)-derived immunostimulatory activity is present in the <3 kDa fraction and quite heat-stable. **(A)** PMNs were incubated for 72 h in the presence or absence of the arginase inhibitor INCB001158 (100 μM). The PMN supernatant (PMN-SN) was harvested (total) and also further subjected to step-wise ultrafiltration with molecular weight cut off filters of 10 kDa and 3 kDa. T cells were stimulated with anti-CD3/anti-CD28-tagged beads and cultured in the different PMN-SNs (+10% dialyzed FCS) for 48 h. T cell proliferation was then determined by [^3^H]thymidine incorporation over 16 h (n=3) and values of stimulated cells in the presence of arginine were set as 100%. Statistical analysis refers to the control conditions of activated T cells in the presence of L-arginine and INCB001158. Statistical calculations were performed with one-way ANOVA and Tukey´s *post hoc* test (*p < 0.05). **(B)** PMNs were incubated for 72 h in the presence or absence of the arginase inhibitor nor-NOHA (1 mM). The PMN supernatant (PMN-SN) was harvested and further incubated for 30 min at different temperatures, as indicated. As controls, normal RPMI cell culture media were treated accordingly. T cells were then stimulated with anti-CD3/anti-CD28-tagged beads and cultured in the different control media or PMN-SNs for 48 h. T cell proliferation was then determined by [^3^H]thymidine incorporation over 16 h (n=3) and the values of stimulated cells in control medium at 37°C were set as 100%. Statistical analysis refers to the hyperactivated T cells in PMN-SN in the presence of nor-NOHA at 37°C. Statistical calculations were performed with one-way ANOVA and Tukey´s *post hoc* test (** p < 0.01, *p < 0.05).

Lastly, we have started to characterize the potent T cell hyperactivating factor(s) within the PMN-SN. In order to get a first idea of the molecular size of the hyperactivating factor(s), we separated newly generated 72 h PMN-SNs into three molecular weight subfractions *via* size exclusion ultrafiltrations with MWCO filters. The resulting three subfractions (> 10 kDa, 3–10 kDa, < 3 kDa) were then used for the T cell proliferation assays and compared with the unseparated PMN-SN in terms of T cell stimulatory potential. In three separate experiments it became apparent that the T cell hyperactivating factor(s) was/were uniformly present in the < 3 kDa subfraction (**Figure 9A**). We also analyzed the heat stability of the hyperactivating factor(s). Newly generated 72 h PMN-SNs (with or without the presence of nor-NOHA) were heated for 30 min at different temperatures, ranging from physiological 37°C to 95°C. After equilibration of all media or PMN-SNs to 37°C, T cells were then stimulated in these different PMN-SNs or cultured in control normal cell culture media, heated accordingly in advance. These experiments demonstrated that the PMN-SN induced hyperactivation was not compromised even by heating at 75°C, while conventional T cell activation was already suppressed when T cells were activated in such preheated cell culture media. At higher preincubation temperatures, conventional T cell activation was completely inhibited, and also T cell hyperactivation was severely compromised (**Figure 9B**).

## Discussion

T cell activation *in vitro* in the context of actively secreted or passively liberated PMN-derived proteins or small molecules is likely reflecting an important aspect of adaptive immunity in the cancer micromilieu *in vivo*. PMN are found at varying degrees in the stroma of diverse cancer entities (18, 27–29) and the interaction of influxing PMN with T cells within the tumor microenvironment can be a critical determinant of tumor growth. Both, pro- and anti-tumoral roles for tumor-associated PMN have been described in a variety of murine tumor models (30) as well as in human cancer patients (18, 27, 31). One important component of PMN- or G-MDSC-based tumor immune evasion is an arginase-mediated arginine depletion (28, 32). In murine tumor models, arginase inhibition fosters T cell infiltration into the tumor stroma (27), leads to enhanced anti-tumoral T cell and NK cell immune reactions (28) and consequently diminished tumor growth (32). A direct interaction of CD66b^+^ PMN with T cells and a correlation of PMN density with suppressed T cell effector functions were recently also demonstrated in human cancer tissue (29). In contrast, tumor-associated PMN can also have opposite consequences in cancer patients: CD66b^+^ PMN density in colorectal cancer stroma is correlated with longer overall survival (33) and a PMN subset in lung cancer presents antigens to T cells and fosters their activation and anti-tumor function (34). These contradictory results might reflect differences of the regulatory role of PMN and G-MDSC in each analyzed tumor model or cancer entity (30), variations in the activation state of the analyzed granulocytic cell type (35, 36) or inborn species differences of activating and suppressive pathways or molecules in granulocytic cells of mice and men (2, 37).

Our work has focused on PMN, the largest physiological population of granulocytic cells in healthy humans, and set up a defined and reproducible experimental system with *in vitro* PMN:T cell ratios that reflect *in vivo* reality (29) to dissect potential T cell stimulatory and/or inhibitory features of PMN. PMN arginase-mediated profound T cell suppression has been amply demonstrated (32). So far, application of an arginase inhibitor in the setting of PMN- or G-MDSC-mediated T cell suppression always reconstituted T cell proliferation to a control level which was achieved by stimulating the T cells in the absence of the granulocytic cells (24, 31, 38, 39). The main finding of our current work is indeed novel and surprising: PMN-mediated T cell suppression is not only prevented by arginase inhibition, but a pronounced hyperactivation of T cells is induced when arginine availability is secured. Why has such T cell hyperactivation not been demonstrated before? We have analyzed T cell functions in PMN-derived supernatant rather than during direct PMN-T cell coculture (16, 31, 33, 39, 40). Other published PMN-SN-mediated activation systems used different T cell stimulation protocols (28) or did not include arginase inhibition during PMN-SN generation as a means to rescue T cell functionality (33). In our own earlier work, which initially unraveled the T cell immunosuppressive activity of PMN arginase 1 (24), we sonicated human PMN after isolation and preincubated cell culture medium with such PMN sonicates of a defined arginase activity with or without arginase inhibitor (24). In such an experimental set-up, we demonstrated that inhibition of T cell activation can be prevented by arginase blockade, but no T cell hyperactivation was detectable. Interestingly, in this earlier work we also tested human pus as an *ex vivo* correlate of dying PMN-dominated inflammation: here a certain degree of T cell hyperactivation was already detectable upon retrospective analysis (24). So far, we can therefore only speculate if the PMN-derived or -secreted immunostimulatory factor is instable, being destroyed by sonication, or if it has to be generated *de novo*, modified or actively processed before release by the PMN. We could already show that the activating factor(s) is/are of a molecular size < 3 kDa (**Figure 9A**) and quite heat-stable (**Figure 9B**). Identification of the hyperstimulatory factor(s) will require dedicated follow-up studies employing mass spectrometry-based approaches. In this context, it was very surprising to detect hyperstimulatory activity in PMN-SN after only 5 min of cell culture preincubation. It has to be noted, however, that a certain amount of PMN activation cannot be prevented in the process of PMN isolation from peripheral blood and it remains unclear if and how this degree of unspecific activation during this procedure contributes to the release of the T cell activating factor(s) in the PMN-SN. PMN granule mobilization can be rapid and granule contents are known to participate in the regulation of T cell immune reactions (41). Very rapid myeloid cell-mediated interference with T cell metabolism has also been shown e.g. for human monocytic MDSC, which can induce profound T cell paralysis after only 30 min of interaction. This suppression is mediated by the MDSC metabolite methylglyoxal, which is transferred from MDSC to interacting T cells already after 10 min, leading to inhibition of T cell mitochondrial respiration, glucose metabolism and, as a consequence, effector functions (42). Interestingly, methylglyoxal reacts with free cytosolic arginine and intracellular arginine of MDSC-paralyzed CD8^+^ T cells was severely depleted (42). In our system, we are confident that arginase inhibition is the prerequisite of T cell hyperactivation since we used two structurally different arginase inhibitors for key experiments and observed comparable T cell hyperactivation (**Figures 1A**, **4** and **8A** and **Supplementary Figure 1**), making unspecific non-target inhibitor effects rather unlikely. Arginase liberation (**Figure 3A**) and concomitant arginine consumption (**Figure 3B**) was detectable during PMN culture *in vitro* without further stimuli-mediated PMN activation, reaffirming our earlier data (24). Also, supplementation of supraphysiological amounts of arginine during T cell culture in PMN-SN prevented T cell inhibition and was associated with a certain degree of T cell hyperactivation (**Figure 3C**). Sufficient arginine availability for T cell function can be achieved by blocking myeloid cell arginase 1 (9, 24) and/or inhibition of T cell mitochondrial arginase 2. The latter strategy was found to enhance metabolic fitness, activation and several effector functions of T cells, but had no impact on their proliferative potential (7, 43). This clearly differs from our data and we also did not see increased proliferation or effector functions in activated T cells upon coincubation with the arginase inhibitor alone. Still, we cannot fully exclude that inhibition of T cell mitochondrial arginase 2 somehow contributed to the T cell hyperactivation: enhanced metabolic fitness might have additively or synergistically enhanced T cell activation features, which become apparent only in the context of the hyperactivating PMN-derived factor(s).

PMN-SN induced hyperstimulation was not globally affecting all T cell functions: while the secretion of most of the tested cytokines was arginine-dependent and the PMN-induced suppression could be prevented by arginase inhibition, a preferentially enhanced secretion was detectable for the cytokines IL-9 and IL-17. In this context it is noticeable that the pleiotropic cytokine IL-17 can direct expansion and chemotactic attraction of PMN into the tumor stroma (44), which would create a positive feedback loop for its own synthesis by PMN-mediated T cell hyperactivation. Also, an earlier and stronger (re-)expression of the T cell activation-associated membrane proteins CD25 and CD28 (**Figure 5**) was detectable, while no such hyperinduction of various negative regulatory membrane proteins was observed (**Figure 6**). This preferential pro-inflammatory T cell membrane protein expression pattern shifts the balance toward costimulation and is therefore potentially relevant for *in vivo* antigen-driven T cell hyperactivation in the setting of cell-cell communication in the cancer microenvironment. In patients with multiple myeloma, arginase I – expressing MDSC are expanded in the peripheral blood and in the bone marrow of patients (36, 45), and T cells are severely compromised in their function (26, 36, 46). Our findings of the highly efficient induction of antigen-specific T cell cytotoxicity (**Figure 7**) as well as the profound stimulation of anergic multiple myeloma patient-derived T cells in the context of arginase inhibition and PMN-SN (**Figure 8**) are potentially highly relevant for clinical application. An arginase inhibitor-based clinical study (EudraCT 2018‐004076‐35) is currently enrolling patients with relapsed or refractory multiple myeloma. Our *in vitro* data clearly support this therapeutic approach to target arginase 1-mediated immunosuppression.

In summary, we have for the first time provided evidence that by inhibiting PMN arginase-induced arginine depletion not only reconstitution of normal T cell function can be achieved but rather a novel hyperactivated T cell phenotype. The identification of the PMN-derived immunostimulatory factor(s) is currently ongoing in order to improve tumor immunotherapies by boosting ineffective or anergic T cells in cancer patients.

## Data Availability Statement

The raw data supporting the conclusions of this article will be made available by the authors, without undue reservation.

## Author Contributions

VV, YB, AW, HE, JW, SI, NS, ST, MT, EC, MM contributed to the conception and/or design of the work. VV, YB, AW, HE, JW, AH, RC, MB contributed to data acquisition. VV, YB, AW, HE, JW, AH, SI, NS, RC, MB, ST, MT, EC, MM performed data analysis and interpretation. VV, YB, MM drafted the manuscript. VV, YB, AW, HE, JW, AH, SI, NS, RC, MB, ST, MT, EC, MM revised the manuscript. This article contains data from the doctoral theses of VV and YB. All authors contributed to the article and approved the submitted version.

## Funding

This work was supported by Deutsche Forschungsgemeinschaft (SFB 1292/1, TP06 to VV, YB, HE, MT, MM; SFB 1292/1, TPZ01 to ST), Calithera and Incyte Corporation.

## Conflict of Interest

SI and NS are employees and equity holders of Incyte Corporation.

The remaining authors declare that the research was conducted in the absence of any commercial or financial relationships that could be construed as a potential conflict of interest.

## Supplementary Material

Supplementary Figure 1 T cell cytokine secretion is hyperactivated by PMN-SN in the presence of arginase inhibition. Human T cells and PMN were isolated from whole blood of healthy donors. PMN were pre-incubated for 72 h in the presence or absence of the arginase inhibitor INCB001158 (100 µM). T cells were stimulated with anti-CD3/anti-CD28-tagged beads in the PMN-SN for 48 h. Cytokines in T cell culture supernatants were analyzed by cytometric bead array (n=6 independent experiments). Unless otherwise stated, statistical analysis refers to the control conditions of activated T cells in the presence of L-arginine and nor-NOHA. Statistical calculations were performed with one-way ANOVA and Tukey´s *post hoc* test (***p < 0.001, **p < 0.01, *p < 0.05).

Supplementary Figure 2 No effect of PMN-SN or arginase inhibition on tumor cell viability. Experimental set-up was as described in **Figure 8**. Results of control conditions for experiments of **Figure 8** in the absence of T cells with or without nor-NOHA are shown.
