# Supplementary figures and images for: Inhibition of Arginase 1 Liberates Potent T Cell Immunostimulatory Activity of Human Neutrophil Granulocytes
Source: Front Immunol. 2021 Feb 26;11:617699. doi: 10.3389/fimmu.2020.617699 (PMC7952869; doi:10.3389/fimmu.2020.617699)

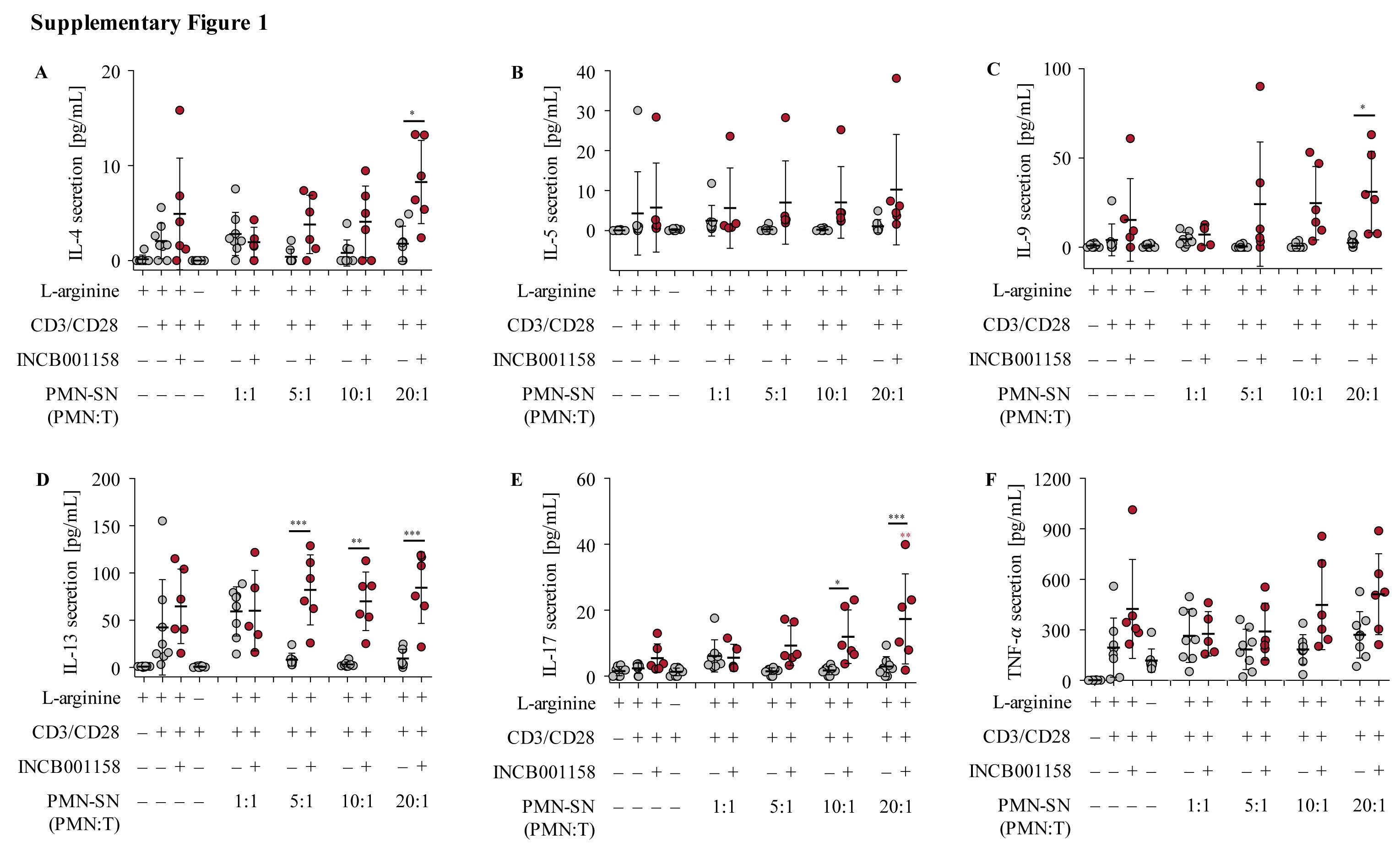

Supplement: Supplementary Figure 1 — T cell cytokine secretion is hyperactivated by PMN-SN in the presence of arginase inhibition. Human T cells and PMN were isolated from whole blood of healthy donors. PMN were pre-incubated for 72 h in the presence or absence of the arginase inhibitor INCB001158 (100 µM). T cells were stimulated with anti-CD3/anti-CD28-tagged beads in the PMN-SN for 48 h. Cytokines in T cell culture supernatants were analyzed by cytometric bead array (n=6 independent experiments). Unless otherwise stated, statistical analysis refers to the control conditions of activated T cells in the presence of L-arginine and nor-NOHA. Statistical calculations were performed with one-way ANOVA and Tukey´s post hoc test (***p < 0.001, **p < 0.01, *p < 0.05). [file Image_1.jpeg]

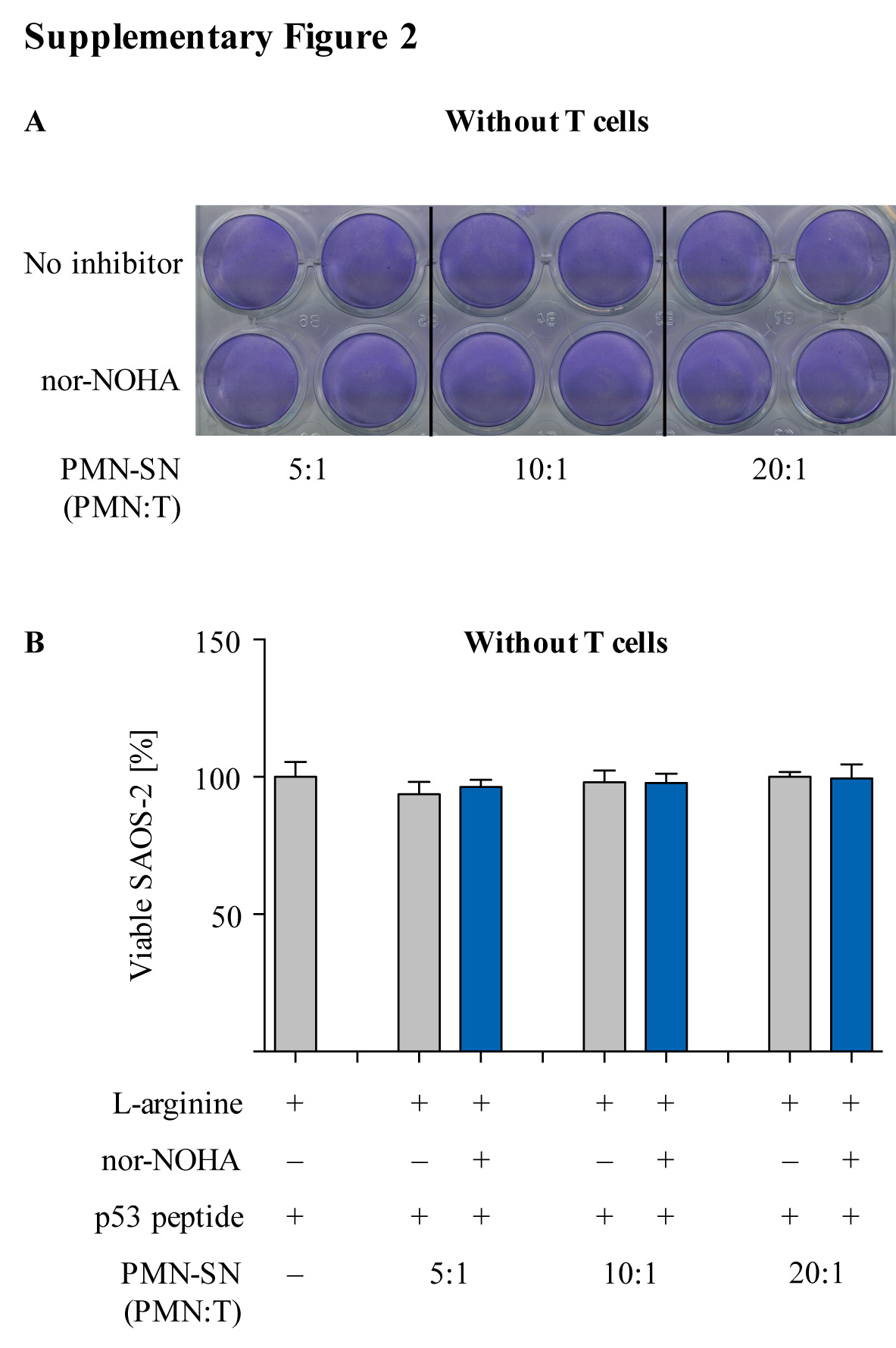

Supplement: Supplementary Figure 2 — No effect of PMN-SN or arginase inhibition on tumor cell viability. Experimental set-up was as described in Figure 8 . Results of control conditions for experiments of Figure 8 in the absence of T cells with or without nor-NOHA are shown. [file Image_2.jpeg]
